# 2513 Evaluation of the current status of urologic training programs in the delivery of transgender care

**DOI:** 10.1017/cts.2018.209

**Published:** 2018-11-21

**Authors:** Daniel Schoenfeld, Beth Drzewiecki

**Affiliations:** 1 Department of Urology, Albert Einstein College of Medicine and Montefiore Medical Center

## Abstract

OBJECTIVES/SPECIFIC AIMS: Transgender individuals remain an underserved population with a unique set of healthcare needs. Given the recent increase in demand for gender affirmation surgery, there is a need to train urologists in the various aspects of surgical management of transgender patients. It is unclear how many urologic residency programs are participating in transgender care. In this study, we sought to determine the current status of urologic training programs in the delivery of transgender care and the sentiments regarding the current and future need to train urologists. METHODS/STUDY POPULATION: Between June and August 2017, a 22 item cross-sectional survey was emailed to all 138 program directors (PDs) as listed by the ACGME. Participation was voluntary and responses were anonymous. Statistical analysis was performed using SAS version 9.4. RESULTS/ANTICIPATED RESULTS: In total, 48 PDs completed the survey (36% of US PDs) and 1 declined to participate. All AUA regions had at least 25% representation, except the Western region (13%). In total, 42% of urology programs that responded participate in institutional transgender health programs; 76% of PDs believe there is a current or future need to train urology residents in the surgical care of transgender patients. PDs were significantly more likely to endorse a need for transgender training if their institution has a transgender health program (95% vs. 58%, *p*<0.005). Similarly, expressed interest in transgender care by trainees was associated with increased belief among PDs in the need for transgender training (95% vs. 58%, *p*<0.005). There was also an association between the presence of a transgender health program and trainee interest in transgender care (64% vs. 33%, *p*=0.04). Need for resident training in the following procedures was cited most often by PDs: complicated catheter placement (91%), orchiectomy (89%), urethral fistula repair (82%), penile/testicle prosthesis insertion (77%), phalloplasty (69%), vaginoplasty (66%), and metoidioplasty/urethral lengthening (54%). Despite the overall consensus that residents should be trained in transgender care, 83% of PDs responded that urologic transgender surgery should be trained in fellowship rather. DISCUSSION/SIGNIFICANCE OF IMPACT: There is an increased demand for surgeons competent in providing gender affirmation surgery. The majority of urology residency PDs believe in the need to train residents in the surgical care of transgender patients. A formalized curriculum for the urologic management of transgender patients should be instituted across residency programs to ensure adequate exposure and competency.

